# Predictors of unsuccessful tuberculosis treatment outcome in **B**hutan: A retrospective study using comprehensive national tuberculosis surveillance data

**DOI:** 10.1038/s41598-026-38023-7

**Published:** 2026-02-06

**Authors:** Thinley Dorji, Karchung Tshering, Lila Adhikari, Thinley Jamtsho, Pavitra Bhujel, Pema Lhaden, Norelle L. Sherry, Chantel Lin, Justin T. Denholm, Sonam Wangchuk, Kristy Horan, Benjamin P. Howden, Patiyan Andersson

**Affiliations:** 1 Kanglung Hospital, Ministry of Health, Trashigang, Bhutan; 2 Department of Microbiology and Immunology at The Peter Doherty Institute for Infection and Immunity, University of Melbourne, Melbourne, Victoria Australia; 3 National Tuberculosis Reference Laboratory, Royal Centre for Disease Control, Ministry of Health, Serbithang, Thimphu, Bhutan; 4 Royal Centre for Disease Control, Ministry of Health, Serbithang, Thimphu, Bhutan; 5 Microbiological Diagnostic Unit, Public Health Laboratory, Department of Microbiology and Immunology at The Peter Doherty Institute for Infection and Immunity, University of Melbourne, Melbourne, Victoria Australia; 6 Department of Infectious Diseases & Immunology, Austin Health, Heidelberg, Victoria, Australia; 7 Royal Melbourne Hospital, Melbourne, Victoria Australia; 8 Victorian Tuberculosis Program, Melbourne Health at the Peter Doherty Institute for Infection and Immunity, University of Melbourne, Melbourne, Victoria Australia; 9 Department of Infectious Diseases at The Peter Doherty Institute for Infection and Immunity, University of Melbourne, Melbourne, Victoria Australia; 10 Centre for Pathogen Genomics, University of Melbourne, Melbourne, Victoria Australia

**Keywords:** Tuberculosis, Bhutan, Treatment outcome, Unsuccessful outcome, Diseases, Health care, Medical research

## Abstract

Despite the high burden of tuberculosis in Bhutan, data on treatment outcomes are limited. Using a national TB dataset from 2018 to 2021, provided by the National Tuberculosis Reference Laboratory, we conducted a retrospective cohort study to assess treatment outcomes and explore factors associated with unsuccessful TB outcomes. Of the 3,619 patients who received TB treatment, 3,330 had a recorded treatment outcome. Among these, 96.2% (*n* = 3,202) had a successful outcome (44.4% (*n* = 1,480) were cured and 51.7% (*n* = 1,722) completed treatment), and 3.8% (*n* = 128) had an unsuccessful outcome (2.8% (*n* = 93) died, 0.7% (*n* = 23) experienced treatment failure, and 0.4% (*n* = 12) were lost to follow-up). Multivariable logistic regression showed patients older than 60 years of age (aOR 4.3; 95% CI 2.09-10.0; p-value < 0.001), diagnosed in 2019 (aOR 1.7; 95% CI 1.03–2.86; p-value 0.041), 2021 (aOR 1.74; 95% CI 1.03–2.97; p-value 0.038), and pulmonary TB (aOR 2.69; 95% CI 1.76–4.27; p-value < 0.001) were more likely to have an unsuccessful treatment outcome. The proportion of successful TB treatment exceeded the global target rate of 90%, with a low rate of unsuccessful outcomes. Patients with pulmonary TB and elderly patients require active follow-up to ensure their treatment success increases. In-depth studies need to be conducted to understand the circumstances leading to the treatment failures and death.

**Supplementary Information:**

The online version contains supplementary material available at 10.1038/s41598-026-38023-7.

## Introduction

Tuberculosis (TB) remains a leading cause of death from a single infectious agent, especially in developing countries. An estimated 23% of the global population has latent TB^1^, with 5–10% of them expected to develop active disease over their lifetime^2^. Approximately 10.7 million TB cases were recorded globally in 2024, with the majority of them in Southeast Asia (34%), Western Pacific (27%) and Africa (25%)^3^.

TB is a public health problem in Bhutan with a prevalence rate of 190/100,000^4^. Globally, TB elimination is hampered by its long treatment duration of at least six months, propensity to develop drug resistance, and latency^5^. Consequently, TB elimination is only possible through early detection and treatment with effective drugs to interrupt the transmission chain. The National Tuberculosis Control Program (NTCP) in Bhutan monitors the treatment outcome of all TB cases to ensure that patients receive timely treatment and are confirmed to be successfully cured. A high proportion of patients with good treatment outcomes reflects the effectiveness of the TB control program^6^, and the use of the World Health Organisation (WHO) standardised definitions facilitates the comparison of TB treatment strategies across countries^7^. NTCP has adopted the WHO End-TB Strategy, which aims to reduce the TB incidence by 90% and TB deaths by 95% by 2035^4^.

Currently, there are few published studies assessing TB treatment outcome in Bhutan, and those available are limited to single health centres^8–10^. Therefore, using comprehensive national TB notification data from 2018 to 2021, we aimed to: (1) evaluate the treatment outcome for all types of TB in Bhutan, and (2) identify the factors associated with unsuccessful treatment outcomes.

## Methods

### Settings

Health care services for the 20 districts in Bhutan are divided into three regions: Eastern, Western and Central region referral centres. At present, all the health services are provided free of cost by the Royal Government of Bhutan, and there are no private health centres in Bhutan. Any presumptive TB patients undergo acid-fast microscopy along with chest X-ray in the local health centres. The sputum smears positive TB cases, and highly suspicious smear-negative TB cases undergo Xpert MTB/RIF for rifampicin resistance and detection of *Mycobacterium tuberculosis* (*Mtb*) bacilli. The smear-positive samples are shipped to the National Tuberculosis Reference Laboratory (NTRL) at the Royal Centre for Disease Control (RCDC), under the Ministry of Health, for routine TB culture and phenotypic testing using the mycobacteria growth indicator tube (MGIT). In addition, all culture-positive samples are tested with the Genotype MTBDRplus assay to detect rifampicin and isoniazid resistance. Samples resistant to rifampicin or both are further tested using the Genotype MTBDRsl assay for second-line drug resistance. TB is a notifiable disease with mandatory reporting in the Tuberculosis Information Surveillance System (TBISS) before treatment commencement. This is a web-based surveillance system managed by NTCP and NTRL. Laboratory and epidemiological data for TB cases are recorded in this system by health workers from all TB centres. TB cases were categorised as pulmonary TB, when the disease involved the lungs and extrapulmonary tuberculosis (EPTB) when an organ other than the lungs was affected. Cases with involvement of both the lungs and other organs were classified as pulmonary TB. The TB cases are treated as per the National Tuberculosis Treatment Guidelines, which are aligned with the WHO treatment recommendations^11^. The current treatment regimen includes six months of rifampicin, isoniazid, ethambutol and pyrazinamide for drug-sensitive TB (DS-TB), given as fixed-dose combinations with two weeks of isolation in the hospital. Multidrug-resistant TB (MDR-TB) are treated with a combination of bedaquiline, pretomanid, linezolid and moxifloxacin and isolated until culture conversion. After discharge, patients are referred to the nearest TB focal persons in the respective hospitals with TB cards. During this time, TB medications are self-administered or provided as family directly observed treatment (DOT), and patients visit the hospitals monthly for drug refills. If a patient fails to attend their monthly scheduled visit, the TB focal person follows up by phone calls.

### Study design and population

This retrospective cohort study was conducted in 2025 and included all TB cases diagnosed and recorded in TBISS from 1 January 2018 to 31 December 2021. The analysis was conducted for this period, as the de-identified data were available only for these years under the permission provided by the Ministry of Health and approval from the ethical clearance committee. Patient information was de-identified by the data custodians at RCDC before being provided to the research team for analysis. Census data were obtained from the National Statistics Bureau (https://www.nsb.gov.bt/publications/annual-dzongkhag-statistics/) and the World Bank database for the estimation of the incidence of the TB during the study period.

### Inclusion and exclusion criteria

We included all TB patients registered for treatment and recorded in the TBISS. In instances where patients were lost to follow-up or treatment failed and re-treated in the same year, they were counted only once, with only the final treatment outcome recorded to avoid duplication. In the final analysis, patients whose treatment outcome was not known or recorded were excluded.

### Case definition

A successful treatment outcome was defined as a patient with TB who was cured or had completed treatment (Table 1)^12^. An unsuccessful treatment outcome encompassed patients whose treatment failed, died, or were lost to follow-up. Since EPTB cases are diagnosed either clinically or through biopsy, treatment outcomes are determined by the physicians based on the clinical assessment into treatment failure or treatment completed. A DS-TB refers to a case in which *Mtb* is susceptible to all first-line TB drugs on Xpert MTB/RIF, line probe assay or by culture-phenotypic drug susceptibility testing. Any DR-TB refers to any case where *Mtb* is resistant to one or more first-line TB drugs, and the patient is managed as DR-TB as per the National Tuberculosis Treatment guideline.

**Table 1:** Outcome definitions used in this paper.

Outcome	Definition
Cured	A case of pulmonary TB who completed the treatment and confirmed to be bacteriologically negative in the last month of treatment or at least one previous occasion.
Treatment completed	A patient who completed the full course of treatment without evidence of failure, but without laboratory confirmation of smear in the last month of treatment or on completion of treatment.
Treatment failure	A patient who is smear positive or culture positive at five months of treatment or later.
Died	Patients with TB who died of any reason during or before treatment.
Lost to follow-up	Patients with TB who interrupted treatment for two or more consecutive months.

### Variables used

The dependent variable was TB treatment outcome - dichotomously classified as successful or unsuccessful. The independent variables included for analysis were patient age, sex, region, year of diagnosis, site of infection, and treatment history.

### Epidemiological data analysis

All statistical analyses were performed using R 4.5.0. Frequencies and proportions were calculated for all the categorical variables. We used the Wilcoxon Rank-Sum test to compare age differences by sex, and the Cochran-Armitage test to assess trends in successful treatment outcomes by year. Kruskal-Wallis test was performed, followed by Dunn’s post hoc test with Bonferroni correction to test for differences in age by treatment outcome. Association of the dichotomous dependent variable with various indicators were assessed in a multivariable logistic regression model using glm. We used the stats::step() function in base R with the backward elimination method, which relies on the Akaike Information Criterion (AIC), to derive a parsimonious model. Backward elimination is preferred over forward and stepwise selection due to its ability to “assess the joint predictive ability of variables”^13^. The parsimonious model was chosen over the complex model to reduce overfitting^13^. The model fit was tested with the likelihood ratio test and Wald test, which showed no significant difference between the full model and the reduced model, supporting the parsimonious model. The strength of associations is presented as odds ratios with a 95% confidence interval. The level of significance was set at a p-value less than 0.05. In instances where multivariable logistic regression could not be used due to a small number of observations during crosstabulations, we used Firth’s penalised logistic regression^14^. Multicollinearity was tested using the variance inflation factor (VIF), and all variables had VIF < 1.5 and were retained in the analysis. Since DR-TB is associated with poor outcomes^2^, we ran separate regression models. Initially, we ran a multivariable logistic model without segregation. Then, for the latter part, we ran a regression model separately for drug-sensitive TB (DS-TB) and drug-resistant TB (DR-TB).

### Ethics approval

The study was approved by the Research and Ethics Board of Health, Ministry of Health, Bhutan (REBH/Approval/2022/027) and the Human Research Ethics Committee, University of Melbourne, Australia (Approval reference no. 2023-26130-41858-3). A waiver for informed consent was approved by the Research and Ethics Board of Health, Ministry of Health, Bhutan, as the study was conducted retrospectively using de-identified data. All methods involving human participants were performed in accordance with relevant guidelines and regulations as per the Declaration of Helsinki.

## Result

### Demographic and clinical characteristics

During the study period (2018–2021), a total of 3,619 patients received TB treatment. The median age of patients at the time of diagnosis was 28 years (range: 0-103 years), with the majority (64%) falling within the 18–39 years age group. The data also included 20 patients who were under six years of age, including five newborns treated for congenital TB. Females accounted for 52% (*n* = 1,877) of TB cases (Refer to Supplementary Table 1). Male patients were significantly older than female patients, with median ages of 30 years and 26 years, respectively (Wilcoxon rank-sum test, *p* < 0.001). Occupation groups were diverse, with students constituting the largest group (23.6%), followed by farmers (19.4%). In the study period, 33 (0.9%) health workers were also treated for TB. Most cases (*n* = 3,254; 89.9%) were diagnosed with TB for the first time, while 9.7% (*n* = 352) were previously treated for TB. The treatment history for 13 cases was not known (Supplementary Table 1). Based on the site of infection, 61.4% of cases (*n* = 2,222) were pulmonary TB, and 38.6% (*n* = 1,397) were extrapulmonary TB.

### Geographical and Temporal distribution of tuberculosis

**Fig. 1:**
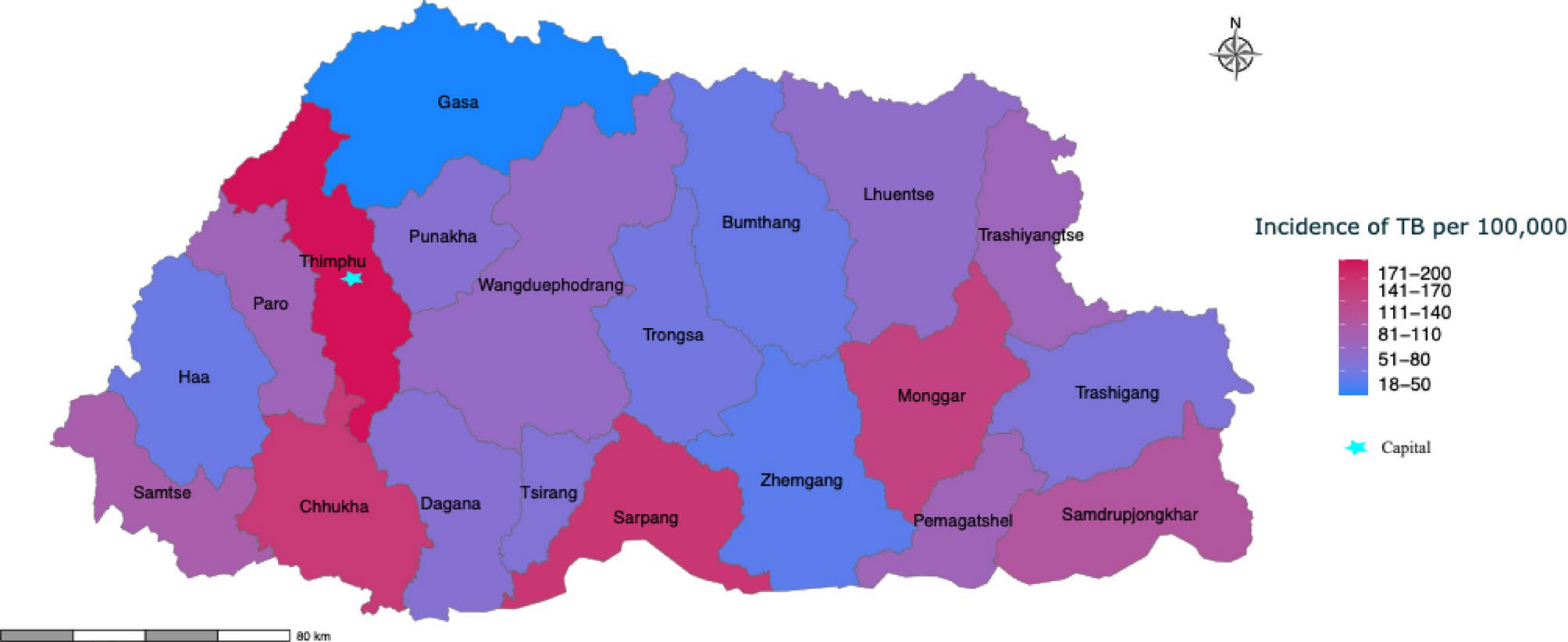
Map of Bhutan illustrating the district-wise distribution of tuberculosis (TB) cases from 2018 to 2021. The star symbol indicates the capital city. Incidence for each district is indicated with a blue (low) to red (high) gradient.

Most TB cases were reported in 2019 (*n* = 981, *n* = 27.1%), followed by 2018 (*n* = 914, 25.3%), while the lowest cases were recorded in 2021 (*n* = 834, 23%). The majority of TB cases were notified from the Western region (82.7%), followed by the Central region (12.5%). The highest number of cases were diagnosed from Thimphu district (36.7%, *n* = 1328), followed by Chhukha (12.2%, *n* = 440), Sarpang district (8.6%, *n* = 311), and Samtse (7%, *n* = 253). The average incidence of TB during the study years was 117.7/100,000 population. After population standardisation, the highest incidence of TB was observed in Thimphu at 192.6/100,000 population, followed by Sarpang (162.9/100,000), Chhukha (158.4/100,000) and Monggar (147.5/100,000) (Fig. 1).

### Treatment outcome based on the testing and resistance status

**Fig. 2:**
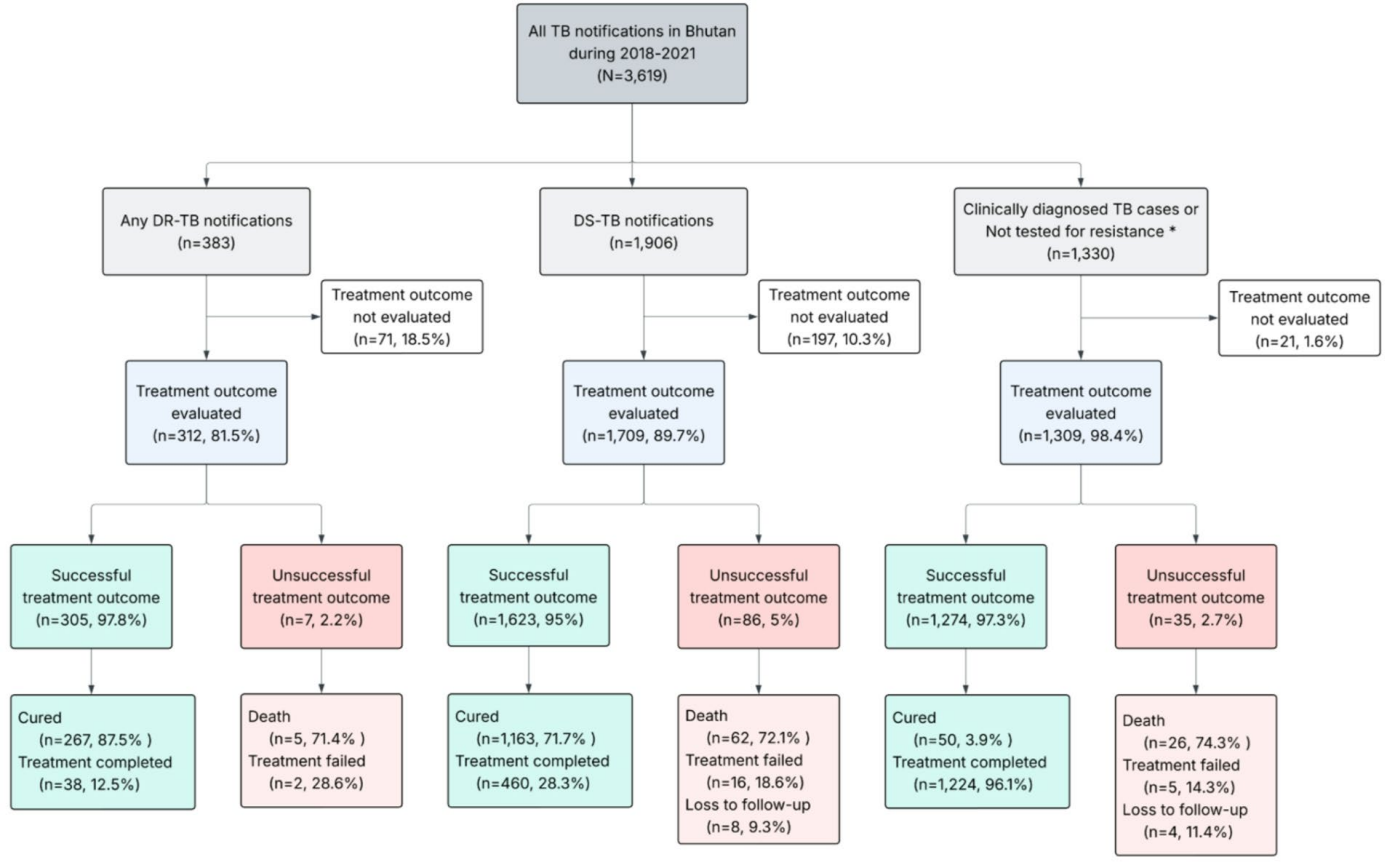
Flowchart depicting treatment outcome for various forms of TB. DR-TB (any drug-resistant TB), DS-TB (drug-sensitive TB). *This also includes bacteriologically confirmed TB cases that were not tested for drug resistance. We have included clinically diagnosed or not tested for resistance for completeness. Please interpret with caution, as we don’t know if these are resistant or sensitive.

For treatment outcome analysis, cases were grouped according to diagnostic and resistance status: drug-sensitive TB, any drug-resistant TB, or clinically diagnosed/not tested TB (Figure. 2). During the study period, 383 cases were resistant to at least one TB drug (rifampicin, isoniazid, ethambutol and streptomycin) based on Xpert MTB/RIF, line probe assay, or phenotypic drug susceptibility testing. Of the DR-TB patients with known treatment outcome (*n* = 312), 84.9% (265/312) were new cases. During the same time, a total of 1,906 cases were diagnosed as DS-TB, while 1,330 cases were clinically diagnosed or not tested for resistance. We had 8% of data (*n* = 289) where the final treatment outcome was not evaluated (Supplementary Table 1). This was predominantly seen during the year 2021, when 14% (*n* = 114) of patients did not have their treatment outcomes evaluated. 15 patients (0.5%) were re-treated in the same year (12 patients failed treatment and three were lost to follow-up) and were included only once in the analysis. The treatment success rate was high across all three groups, ranging from 97.8% for any DR-TB to 95% for DS-TB. Deaths during the TB treatment accounted for most unsuccessful treatment outcomes across all three groups (Fig. 2). Based on the site of infection, the successful outcome varied from 94.9% (*n* = 1,882/1,984) among pulmonary TB patients to 98.1% (1,320/1346) among extrapulmonary TB.

**Fig. 3:**
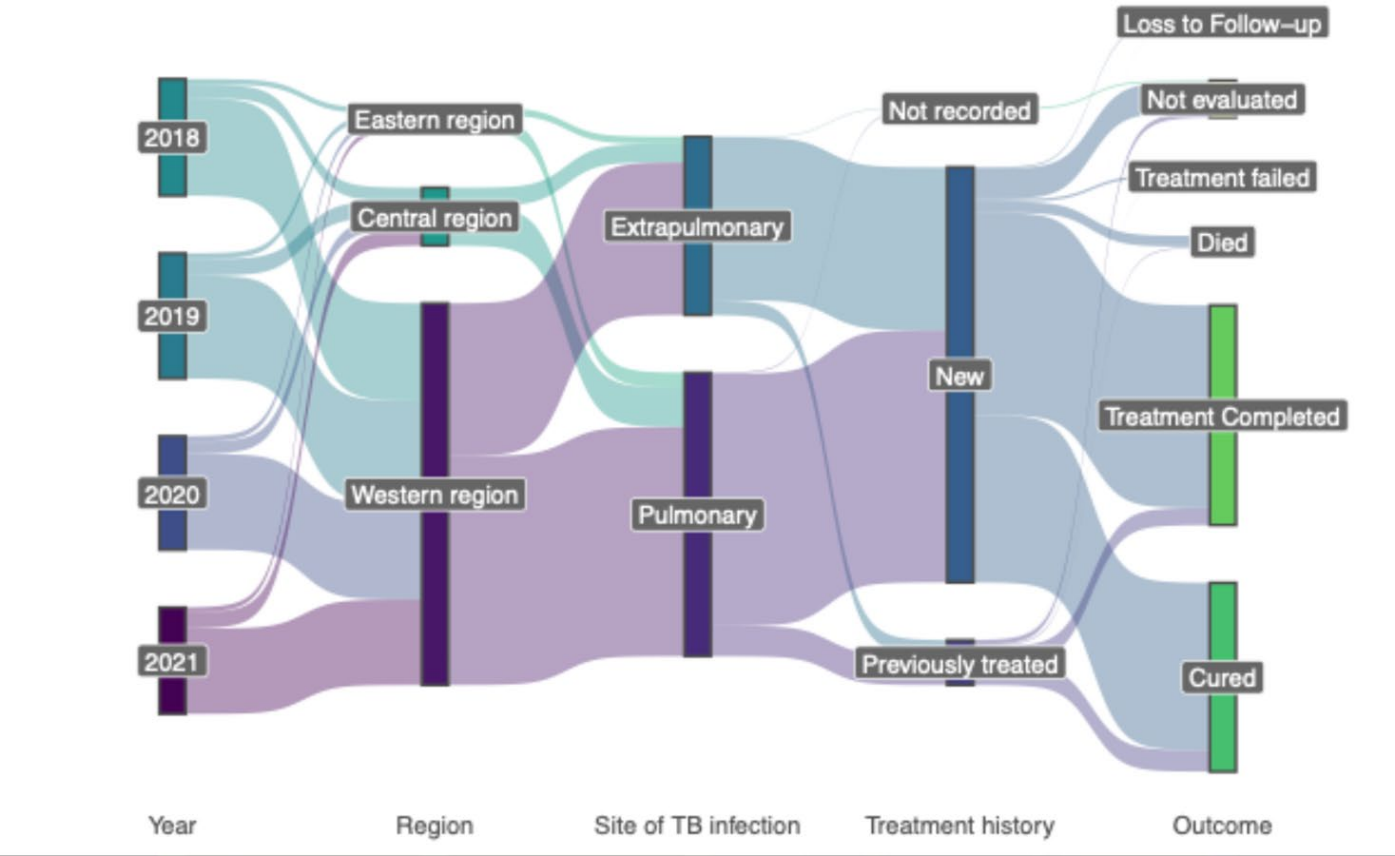
Sankey diagram summarising the TB treatment outcome by year of diagnosis, region, site of infection, and treatment history. It represents the pairwise relationship between the adjacent variables.

Overall, during the study period, 51.7% (*n* = 1722) completed treatment, 44.4% (*n* = 1480) were cured, 2.8% (*n* = 93) died with TB, 0.7% (*n* = 23) failed treatment, and 0.4% (*n* = 12) were lost to follow-up. The proportions of different treatment outcome types varied among different districts (Supplementary Figure. 1). Treatment failures were seen mainly among patients from Bumthang, Dagana and Lhuentse. In contrast, most deaths were seen among patients from Trongsa, Trashigang and TrashiYangtse. The small number of cases in some districts prevented us from conducting further statistical analysis. We further explored the patient’s characteristics by the outcome types (Supplementary Table 1). The median age of patients who died was 54 years (range: 36–69), which was significantly older (*p* < 0.05) than patients with other treatment outcomes, except lost to follow-up. Most patients who died were older than 60 years (46.2%, *n* = 43), diagnosed with pulmonary TB (80.6%, *n* = 75) and were farmers (50.5%, *n* = 47). The overall relationship between the year of diagnosis, region, treatment history and site of infection with treatment outcome is summarised in Fig. 3.

### Determinants of treatment outcome

Considering all available cases (including those whose treatment outcome was not evaluated), the proportion of successful outcomes was 88.5%. After excluding cases without recorded treatment outcomes (*n* = 289, 8% of total cases), the overall treatment success rate increased to 96.2% (95% CI: 95.43% − 96.77%), with no significant variation over time (Cochran-Armitage test; *p* = 0.108).

Table 2 shows the results of univariate and multivariable logistic regression assessing variables associated with unsuccessful outcomes. Patients older than 60 years of age (aOR 4.3; 95% CI 2.09-10; *p* < 0.001), diagnosed in 2019 (aOR 1.7; 95% CI 1.03–2.86; p-value 0.041), 2021 (aOR 1.74; 95% CI 1.03–2.97; *p* = 0.038), and pulmonary TB (aOR 2.69; 95% CI 1.76–4.27; *p* < 0.001) were significantly more likely to have unsuccessful treatment outcome in the multivariable logistic regression. Although male patients were 1.46 times more likely to have an unsuccessful outcome in the univariate analysis, this association was not retained in the multivariable model.

**Table 2:** Determinants of successful treatment outcome in Bhutan.

Characteristic	Proportion of cases with treatment outcome		Unadjusted			Adjusted		
	Successful outcome *N* = 3,202 (96.2%)	Unsuccessful outcome *N* = 128 (3.8%)	OR	95% CI	*p*-value	OR	95% CI	*p*-value
Age (years)								
< 18 years	281 (8.8%)	8 (6.3%)	—	—		—	—	
18–39 years	2,092 (65.3%)	45 (35.2%)	0.76	0.37, 1.75	0.5	0.68	0.33, 1.57	0.3
40–59 years	487 (15.2%)	28 (21.9%)	2.02	0.95, 4.80	0.085	1.88	0.88, 4.49	0.12
≥ 60 years	342 (10.7%)	47 (36.7%)	4.83	2.37, 11.2	< 0.001	4.30	2.09, 10.0	< 0.001
Gender								
Female	1,679 (52.4%)	55 (43.0%)	—	—				
Male	1,523 (47.6%)	73 (57.0%)	1.46	1.03, 2.10	0.037			
Region								
Central region	368 (11.5%)	19 (14.8%)	—	—				
Eastern region	143 (4.5%)	11 (8.6%)	1.49	0.67, 3.16	0.3			
Western region	2,691 (84.0%)	98 (76.6%)	0.71	0.44, 1.20	0.2			
Treatment history								
Previously treated	304 (9.5%)	11 (8.6%)	—	—				
New	2,898 (90.5%)	117 (91.4%)	1.12	0.62, 2.22	0.7			
Year of diagnosis								
2018	822 (25.7%)	26 (20.3%)	—	—		—	—	
2019	880 (27.5%)	42 (32.8%)	1.51	0.92, 2.51	0.11	1.70	1.03, 2.86	0.041
2020	816 (25.5%)	24 (18.8%)	0.93	0.53, 1.64	0.8	0.98	0.55, 1.73	> 0.9
2021	684 (21.4%)	36 (28.1%)	1.66	1.00, 2.81	0.052	1.74	1.03, 2.97	0.038
Site of infection								
Extrapulmonary	1,320 (41.2%)	26 (20.3%)	—	—		—	—	
Pulmonary	1,882 (58.8%)	102 (79.7%)	2.75	1.81, 4.34	< 0.001	2.69	1.76, 4.27	< 0.001

Since drug-resistant TB have poor outcomes, we stratified the data and ran a separate regression model to look for factors associated with unsuccessful treatment outcomes for drug-sensitive TB and any DR-TB cases. We found that patients older than 60 years (aOR 7.8; 95% CI 2.72-33.0, *p* < 0.001) and patients aged 40–59 years (aOR 3.64; 95% CI 1.22-15.7, *p* = 0.04) (Supplementary Table 2) were more likely to have an unsuccessful treatment outcome for drug-sensitive TB. For any drug-resistant TB, we applied Firth bias-reduced logistic regression due to a smaller number of observations (Supplementary Table 3). However, no factors were found to be significantly associated with unsuccessful treatment outcomes.

## Discussion

Using a comprehensive national TB dataset, this study demonstrated a high successful TB treatment outcome in Bhutan. Patients older than 60 years, diagnosed with TB in 2019, 2021, and patients with pulmonary TB were more likely to have an unsuccessful TB treatment outcome.

Excluding incomplete outcome data, the proportion of patients with unsuccessful treatment outcomes in our dataset was low, with the rate of successful outcomes exceeding the global target of 90%^15^. This is consistent with previous studies in Bhutan assessing the treatment outcome at the level of health facilities and among children, which showed a low rate of unsuccessful outcomes^8,9,16^. The low proportion of patients with unsuccessful treatment in this study is similar to studies from China^17^. This is likely due to the provision of free health care and a regular follow-up in Bhutan. Additionally, we also have high BCG vaccination coverage in Bhutan^18^, which is usually associated with good treatment outcomes^19^. Although we did not have data on BCG vaccination, we can postulate that most would have been vaccinated due to high coverage (99%) for more than two decades^18^. Finally, the low prevalence of HIV/AIDS in Bhutan^20^ likely further contributes to the small proportion of unsuccessful TB treatment outcomes^21^. A similar study looking at one of the MDR-TB centres showed high treatment success among the MDR/RR-TB patients^22^. This likely represents the added benefit of hospital isolation, which enables close monitoring and follow-up for adverse reactions during the early phase of treatment. During this period, all MDR-TB patients receive nutritional support from the government. Furthermore, after the discharge, MDR-TB patients undergo monthly sputum smear microscopy and culture, ensuring continued monitoring. This rigorous follow-up promotes drug adherence and prompt management of complications^11^.

Despite the overall low proportion of unsuccessful treatment outcomes, most of these were due to deaths (2.6%). While already lower than the case fatality rate reduction threshold target proposed by WHO (6.5%) by 2025^3^, evidence-based approaches could further reduce these deaths. Although we lacked information on the timing of the patients’ deaths during treatment, previous studies have shown that most deaths occur early in the treatment (< 2 months) and are often associated with pulmonary TB^23^. This highlights the importance of closely monitoring and following up on the pulmonary TB cases during the intensive treatment phase, especially among elderly patients. Delayed TB diagnosis and treatment are linked to higher mortality rates among TB patients^24^. The passive screening approach for TB used in Bhutan could affect timely diagnosis, and delays of 30 days have been reported previously^25^. Increased active TB screening for early diagnosis could have a considerable impact on reducing deaths - it would also likely have a positive effect on decreasing transmission. Non-adherence to the treatment regimen has been shown to increase the mortality rate from TB^26^. However, we do not have any information on adherence. Therefore, further in-depth verbal autopsy can be performed concerning those who died to understand adherence and other risk factors. One proven method of improving drug adherence includes electronic reminders as well as incentives^27^.

A high proportion of TB was observed among students, potentially a consequence of high contact with index cases and indoor transmission of the disease in classrooms and dormitories. Active case screening could be focused on this cohort, particularly in high-burden districts of Bhutan. Furthermore, it is essential to increase awareness around TB symptoms and prevention in these settings, as knowledge about TB in Bhutan remains low^28^. We also observed health workers diagnosed with TB. Although this group accounts for fewer than 1%, their exposure remains a significant infection control concern, particularly due to the risks of transmitting the disease to vulnerable populations during service delivery. Despite evidence of regular screening leading to a decrease in TB among the health workers^29^, there are currently no formal guidelines in place for the screening of health workers for TB in Bhutan^4^.

Patients diagnosed and treated for TB in 2019 and 2021 were more likely to have an unsuccessful outcome. One reason for 2019 could be due to the high number of TB cases in 2019 compared to other years during the study period. Poor outcomes in 2021 were mainly due to disruptions in TB management and care associated with lockdowns and reassignment of health workers to COVID-19 responses during the pandemic^30^. There was a deterioration in record-keeping during the pandemic, with the majority of incomplete data observed in 2021. The TB case detection rate in Bhutan also dropped from 80% in 2019 to 67% in 2021^31^. Poor TB treatment outcomes during the pandemic were also described in other settings^32^, and an increase in TB deaths was observed globally^33^.

Consistent with our findings, cases diagnosed as pulmonary TB are associated with higher rates of unsuccessful outcomes compared to the other forms of TB, likely due to high bacillary load and advanced disease^21,34^. Advanced age has been associated with unsuccessful TB treatment outcomes^21,35^, likely due to a decrease in immunity as well as the development of co-morbidities. However, association with co-morbidities could not be assessed in this study as these data were not available. Therefore, older patients and patients with pulmonary TB should be regularly followed up to ensure smear conversion occurs and have a higher likelihood of cure. In addition, it is essential that active screening for TB is conducted among the elderly population, as some studies have shown a high proportion of asymptomatic cases among this population^36^. The provision of nutritional support would also improve treatment outcomes^37^.

Most of the DR-TB cases in our dataset were new cases, indicating that these cases were likely acquired through transmission rather than *de novo* mutations. The majority of these cases were multidrug-resistant TB^38^, and whole genome sequencing revealed high clustering among the MDR-TB in Bhutan^39^. Despite this, several factors favour Bhutan’s potential to achieve a significant reduction in TB burden. While Bhutan has a high prevalence of TB, the small population means that the absolute number of TB cases is low when compared to other high-burden countries. Further, health care services are provided free of cost, and BCG vaccination is integrated within the expanded immunisation programme. As Bhutan strives towards achieving the End TB strategy, “patient-centered care”^40^ needs to be considered. Currently, pulmonary smear-positive TB cases are admitted to isolation wards for a few weeks to ensure interruption of transmission and patient drug compliance. However, recent evidence has shown no differences between health-worker-administered DOT and self-administered therapy^41^, and advocates for home-based isolation^42^ to prevent untoward mental stress and stigmatisation of the patient^43^. It is unlikely that home-based isolation would further exacerbate household transmission post-diagnosis, as the patient would be on treatment. An added benefit of home isolation is the reduction of catastrophic costs (costs ≥ 20% of household annual income) as well as health system costs. Therefore, health facility isolation should be reserved for severely ill, undernourished patients and those with co-morbid illness, which increases the risk of drug reactions.

To our knowledge, this is the first study assessing the TB treatment outcome at the national level using a comprehensive national TB dataset. Nonetheless, there are some limitations. We lacked information on co-morbid illnesses, risky behaviours (e.g. smoking and alcohol consumption), symptom duration, and drug adherence - factors that could influence treatment outcomes. Routinely collected surveillance data will have varying degrees of completeness. Some important indicators known to influence treatment outcome, such as weight^44^, were not recorded in the online reporting system for the majority of cases and were dropped from the analysis. Approximately 8% of the outcome data were not updated in the system by the health workers and hence had to be excluded during the final analysis. Improved completeness and accuracy of the record keeping would support real-time monitoring and enhance management of TB patients. Further, as disease incidence decreases, more variables could be incorporated in the surveillance system to inform the design and strategies to improve TB treatment adherence. In our dataset, most cases were pulmonary TB, but for a considerable proportion, the treatment outcome was listed as treatment completed rather than cured. Although both cure and treatment show good prognosis, it is best practice for pulmonary TB to be bacteriologically declared negative after completion of treatment^12^. Therefore, testing protocols and subsequent documentation of bacteriologic confirmation of cure need to be enhanced.

## Conclusion

For the first time, we present the treatment outcome rates using comprehensive national data for the years 2018–2021 in Bhutan. The findings from this study can be used to formulate support services and improve monitoring of the patients who are at high risk of treatment failure. Despite Bhutan’s high treatment success rate, there remains potential to further improve treatment outcomes through decreasing TB mortality. Additionally, documentation in the online surveillance system could be strengthened. Our findings were generated using the national data and therefore can be used for inter-country comparison across the region with a similar health system.

## Supplementary Information

Below is the link to the electronic supplementary material.

Supplementary Material 1

## Data Availability

Data requests can be made to the corresponding author, with sharing subject to the discretion of the Royal Centre for Disease Control, Bhutan, as the data custodians.
