# Supplementary material for: Predictors of unsuccessful tuberculosis treatment outcome in Bhutan: A retrospective study using comprehensive national tuberculosis surveillance data
Source: Sci Rep. 2026 Feb 6;16:7535. doi: 10.1038/s41598-026-38023-7 (PMC12932646; doi:10.1038/s41598-026-38023-7)

Predictors of unsuccessful tuberculosis treatment outcome in Bhutan: A retrospective study using comprehensive national tuberculosis surveillance data

Authors: Thinley Dorji[1,2*], Karchung Tshering[3], Lila Adhikari[3], Thinley Jamtsho[4], Pavitra Bhujel[3], Pema Lhaden[3], Norelle L. Sherry[2, 5, 6], Chantel Lin[5], Justin T. Denholm[7, 8, 9], Sonam Wangchuk[4], Kristy Horan[2, 5], Benjamin P. Howden[2, 5, 6, 10 #,], Patiyan Andersson[2,5,* #]

Supplementary Tables

Supplementary Table 1. Sociodemographic and clinical characteristics by treatment outcome types.

Characteristic	Cured N = 1,480	Treatment completed N = 1,722	Treatment failed N = 23	Died N = 93	Loss to follow-up N = 12	Not evaluated N = 289	Overall N = 3,619
**Age (median)**	27	28	29	54	37	27	28
**Age**							
<18 years	101 (6.8%)	180 (10.5%)	4 (17.4%)	3 (3.2%)	1 (8.3%)	30 (10.4%)	319 (8.8%)
18-39 years	1,011 (68.3%)	1,081 (62.8%)	13 (56.5%)	25 (26.9%)	7 (58.3%)	166 (57.4%)	2,303 (63.6%)
40-59 years	219 (14.8%)	268 (15.6%)	3 (13.0%)	22 (23.7%)	3 (25.0%)	64 (22.1%)	579 (16.0%)
≥ 60 years	149 (10.1%)	193 (11.2%)	3 (13.0%)	43 (46.2%)	1 (8.3%)	29 (10.0%)	418 (11.6%)
**Gender**							
Female	748 (50.5%)	931 (54.1%)	12 (52.2%)	40 (43.0%)	3 (25.0%)	143 (49.5%)	1,877 (51.9%)
Male	732 (49.5%)	791 (45.9%)	11 (47.8%)	53 (57.0%)	9 (75.0%)	146 (50.5%)	1,742 (48.1%)
**Treatment history**							
Missing	0 (0.0%)	0 (0.0%)	0 (0.0%)	0 (0.0%)	0 (0.0%)	13 (4.5%)	13 (0.4%)
New	1,311 (88.6%)	1,587 (92.2%)	21 (91.3%)	85 (91.4%)	11 (91.7%)	239 (82.7%)	3,254 (89.9%)
Previously treated	169 (11.4%)	135 (7.8%)	2 (8.7%)	8 (8.6%)	1 (8.3%)	37 (12.8%)	352 (9.7%)
**Region**							

Characteristic	Cured N = 1,480	Treatment completed N = 1,722	Treatment failed N = 23	Died N = 93	Loss to follow-up N = 12	Not evaluated N = 289	Overall N = 3,619
Central region	175 (11.8%)	193 (11.2%)	4 (17.4%)	12 (12.9%)	3 (25.0%)	67 (23.2%)	454 (12.5%)
Eastern region	70 (4.7%)	73 (4.2%)	3 (13.0%)	6 (6.5%)	2 (16.7%)	19 (6.6%)	173 (4.8%)
Western region	1,235 (83.4%)	1,456 (84.6%)	16 (69.6%)	75 (80.6%)	7 (58.3%)	203 (70.2%)	2,992 (82.7%)
**Year of diagnosis**							
2018	368 (24.9%)	454 (26.4%)	6 (26.1%)	19 (20.4%)	1 (8.3%)	66 (22.8%)	914 (25.3%)
2019	430 (29.1%)	450 (26.1%)	9 (39.1%)	28 (30.1%)	5 (41.7%)	59 (20.4%)	981 (27.1%)
2020	379 (25.6%)	437 (25.4%)	3 (13.0%)	20 (21.5%)	1 (8.3%)	50 (17.3%)	890 (24.6%)
2021	303 (20.5%)	381 (22.1%)	5 (21.7%)	26 (28.0%)	5 (41.7%)	114 (39.4%)	834 (23.0%)
**Occupation**							
Armed forces	23 (1.6%)	33 (1.9%)	1 (4.3%)	0 (0.0%)	0 (0.0%)	5 (1.7%)	62 (1.7%)
Civil Servant	76 (5.1%)	119 (6.9%)	0 (0.0%)	3 (3.2%)	0 (0.0%)	11 (3.8%)	209 (5.8%)
Corporate employee	33 (2.2%)	44 (2.6%)	0 (0.0%)	0 (0.0%)	0 (0.0%)	7 (2.4%)	84 (2.3%)
Dependent	15 (1.0%)	24 (1.4%)	0 (0.0%)	1 (1.1%)	0 (0.0%)	1 (0.3%)	41 (1.1%)
Driver	25 (1.7%)	31 (1.8%)	1 (4.3%)	1 (1.1%)	1 (8.3%)	3 (1.0%)	62 (1.7%)

Characteristic	Cured N = 1,480	Treatment completed N = 1,722	Treatment failed N = 23	Died N = 93	Loss to follow-up N = 12	Not evaluated N = 289	Overall N = 3,619
Farmer	257 (17.4%)	312 (18.1%)	6 (26.1%)	47 (50.5%)	3 (25.0%)	78 (27.0%)	703 (19.4%)
Housewife	152 (10.3%)	190 (11.0%)	3 (13.0%)	7 (7.5%)	0 (0.0%)	30 (10.4%)	382 (10.6%)
Labour	3 (0.2%)	1 (0.1%)	0 (0.0%)	0 (0.0%)	2 (16.7%)	3 (1.0%)	9 (0.2%)
Minors	0 (0.0%)	26 (1.5%)	0 (0.0%)	2 (2.2%)	0 (0.0%)	0 (0.0%)	28 (0.8%)
Monk/Nun	19 (1.3%)	31 (1.8%)	0 (0.0%)	1 (1.1%)	1 (8.3%)	4 (1.4%)	56 (1.5%)
Others	272 (18.4%)	223 (13.0%)	3 (13.0%)	5 (5.4%)	1 (8.3%)	45 (15.6%)	549 (15.2%)
Prisoner	11 (0.7%)	20 (1.2%)	0 (0.0%)	0 (0.0%)	0 (0.0%)	1 (0.3%)	32 (0.9%)
Private /Business	196 (13.2%)	205 (11.9%)	1 (4.3%)	2 (2.2%)	2 (16.7%)	26 (9.0%)	432 (11.9%)
Retiree	53 (3.6%)	40 (2.3%)	2 (8.7%)	17 (18.3%)	0 (0.0%)	4 (1.4%)	116 (3.2%)
Student/Trainee	345 (23.3%)	423 (24.6%)	6 (26.1%)	7 (7.5%)	2 (16.7%)	71 (24.6%)	854 (23.6%)
**Site of infection**							
Extrapulmonary	0 (0.0%)	1,320 (76.7%)	5 (21.7%)	18 (19.4%)	3 (25.0%)	51 (17.6%)	1,397 (38.6%)
Pulmonary	1,480 (100.0%)	402 (23.3%)	18 (78.3%)	75 (80.6%)	9 (75.0%)	238 (82.4%)	2,222 (61.4%)

Supplementary Table 2. Determinants of unsuccessful treatment outcome among DS-TB in Bhutan.

	Proportion of drug-sensitive TB cases with treatment outcome		Unadjusted			Adjusted		
**Characteristic**	**Successful outcome** N = 1,623 (95%)	**Unsuccessful outcome** N = 86 (5%)	**OR**	**95% CI**	**p-value**	**OR**	**95% CI**	**p-value**
**Age (years)**								
<18 years	117 (7.2%)	3 (3.5%)	—	—		—	—	
18-39 years	1,127 (69.4%)	30 (34.9%)	1.04	0.36, 4.38	>0.9	1.04	0.36, 4.38	>0.9
40-59 years	214 (13.2%)	20 (23.3%)	3.64	1.22, 15.7	0.040	3.64	1.22, 15.7	0.040
≥ 60 years	165 (10.2%)	33 (38.4%)	7.80	2.72, 33.0	<0.001	7.80	2.72, 33.0	<0.001
**Gender**								
Female	863 (53.2%)	36 (41.9%)	—	—				
Male	760 (46.8%)	50 (58.1%)	1.58	1.02, 2.46	0.042			
**Region**								
Central region	175 (10.8%)	12 (14.0%)	—	—				
Eastern region	67 (4.1%)	6 (7.0%)	1.31	0.44, 3.51	0.6			
Western region	1,381 (85.1%)	68 (79.1%)	0.72	0.40, 1.42	0.3			
**Treatment history**								
New	1,449 (89.3%)	78 (90.7%)	1.17	0.59, 2.67	0.7			
Previously treated	174 (10.7%)	8 (9.3%)	—	—				
**Year of diagnosis**								
2018	382 (23.5%)	14 (16.3%)	—	—				
2019	413 (25.4%)	22 (25.6%)	1.45	0.74, 2.95	0.3			
2020	443 (27.3%)	22 (25.6%)	1.36	0.69, 2.75	0.4			
2021	385 (23.7%)	28 (32.6%)	1.98	1.05, 3.93	0.041			

	Proportion of drug-sensitive TB cases with treatment outcome		Unadjusted			Adjusted		
**Characteristic**	**Successful outcome** N = 1,623 (95%)	**Unsuccessful outcome** N = 86 (5%)	**OR**	**95% CI**	**p-value**	**OR**	**95% CI**	**p-value**

Abbreviations: CI = Confidence Interval, OR = Odds Ratio

Supplementary Table 3. Determinants of successful treatment outcome among any drug-resistant TB in Bhutan.

	Proportion of any drug-resistant TB cases with treatment outcome		Unadjusted			Adjusted		
**Characteristic**	**Successful outcome** N = 305 (97.8%)	**Unsuccessful outcome** N = 7 (2.2%)	**OR**	**95% CI**	**p-value**	**OR**	**95% CI**	**p-value**
**Age (years)**								
<18 years	17 (5.6%)	1 (14%)	—	—				
18-39 years	225 (74%)	4 (57%)	0.233	0.04, 2.41	0.186	0.339	0.05, 3.70	0.323
40-59 years	46 (15%)	1 (14%)	0.376	0.029, 4.87	0.418	0.791	0.05, 12.04	0.84
≥ 60 years	17 (5.6%)	1 (14%)	1.00	0.076, 13.2	1	2.29	0.12, 46.05	0.561
**Sex**								
Female	156 (51%)	5 (71%)	—	—				
Male	149 (49%)	2 (29%)	0.476	0.085, 2.01	0.319	0.346	0.047, 1.779	0.212
**Region**								
Central region	35 (11%)	2 (29%)	—	—				
Eastern region	13 (4.3%)	0 (0%)	0.526	0.004, 7.03	0.666	0.643	0.004, 10.86	0.782
Western region	257 (84%)	5 (71%)	0.303	0.07, 1.74	0.161	0.251	0.053, 1.485	0.117
**Treatment history**								
New	258 (85%)	7 (100%)	2.76	0.326, 360.	0.424	1.96	0.215, 257.38	0.62
Previously Treated	47 (15%)	0 (0%)						
**Year of diagnosis**								
2018	85 (28%)	1 (14%)	—	—				
2019	104 (34%)	3 (43%)	1.91	0.307, 20.0	0.498	2	0.316, 21.15	0.468
2020	62 (20%)	0 (0%)	0.456	0.003, 8.69	0.613	0.443	0.003, 8.17	0.597

	Proportion of any drug-resistant TB cases with treatment outcome		Unadjusted			Adjusted		
**Characteristic**	**Successful outcome** N = 305 (97.8%)	**Unsuccessful outcome** N = 7 (2.2%)	**OR**	**95% CI**	**p-value**	**OR**	**95% CI**	**p-value**
2021	54 (18%)	3 (43%)	3.66	0.584, 38.5	0.168	3.4	0.539, 35.25	0.195

Due to small sample size and rare events, we used Firth's penalized logistic regression using the r-package logistf.

Supplementary Figures

Supplementary Figure 1. Distribution of treatment outcomes stratified by district in Bhutan.

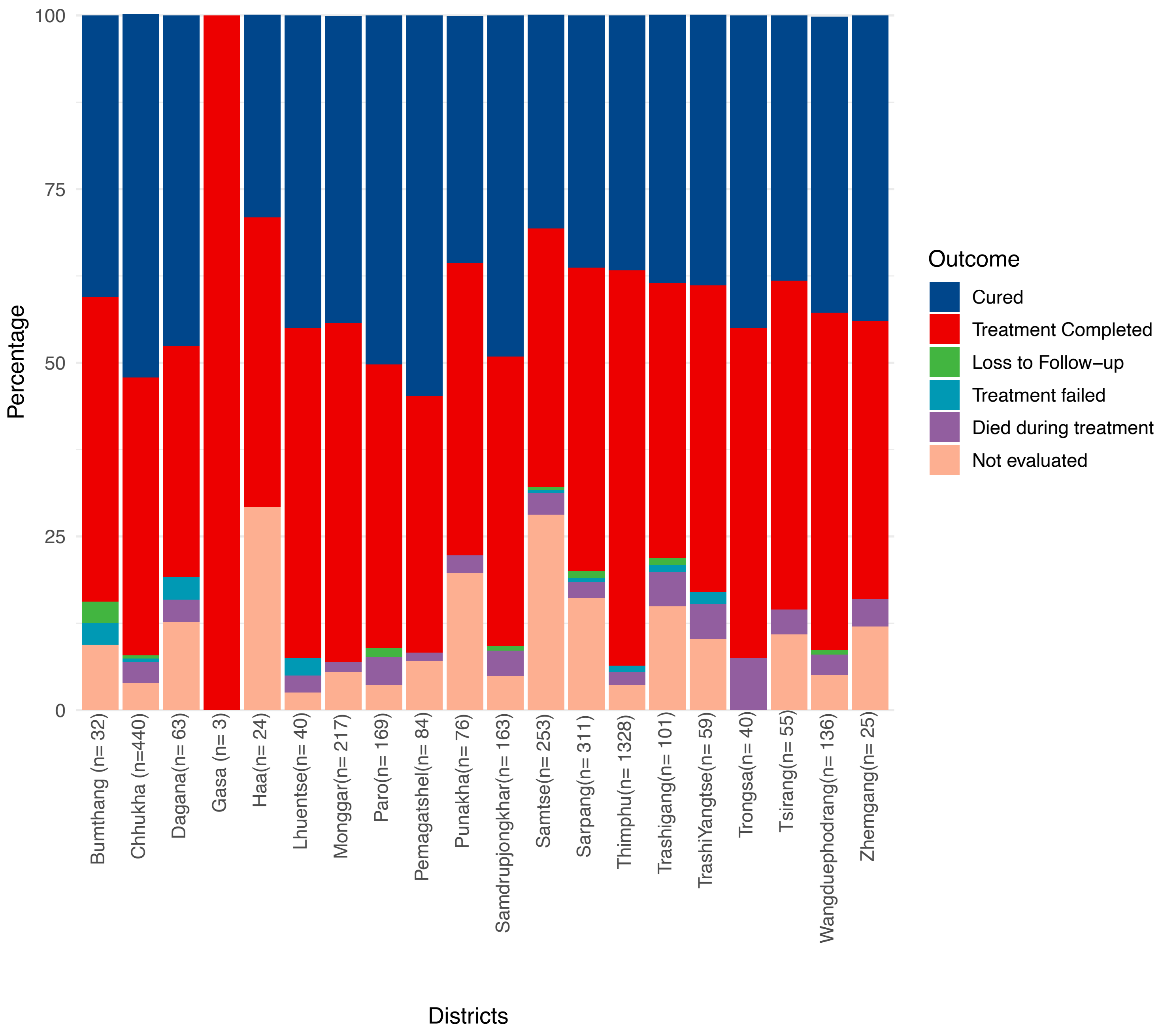

Supplement: Supplementary file 1 — Supplementary Material 1 [file 41598_2026_38023_MOESM1_ESM.pdf]
